# Effects of Scaffolds on Urine- and Urothelial Carcinoma Tissue-Derived Organoids from Bladder Cancer Patients

**DOI:** 10.3390/cells12162108

**Published:** 2023-08-20

**Authors:** Simon Walz, Paul Pollehne, Philipp Vollmer, Wilhelm K. Aicher, Arnulf Stenzl, Niklas Harland, Bastian Amend

**Affiliations:** 1 Department of Urology, University of Tuebingen Hospital, 72076 Tübingen, Germany; 2 Center for Medical Research, University of Tuebingen, 72074 Tübingen, Germany

**Keywords:** bladder cancer organoids, scaffold materials, Matrigel^®^, basement membrane extract, GrowDex^®^

## Abstract

Organoids are three-dimensional constructs generated by placing cells in scaffolds to facilitate the growth of cultures with cell–cell and cell–matrix interactions close to the in vivo situation. Organoids may contain different types of cells, including cancer cells, progenitor cells, or differentiated cells. As distinct culture conditions have significant effects on cell metabolism, we explored the expansion of cells and expression of marker genes in bladder cancer cells expanded in two different common scaffolds. The cells were seeded in basement membrane extract (BME; s.c., Matrigel^®^) or in a cellulose-derived hydrogel (GrowDex^®^, GD) and cultured. The size of organoids and expression of marker genes were studied. We discovered that BME facilitated the growth of significantly larger organoids of cancer cell line RT112 (*p* < 0.05), cells from a solid tumor (*p* < 0.001), and a voiding urine sample (*p* < 0.001). Expression of proliferation marker Ki76, transcription factor TP63, cytokeratin CK20, and cell surface marker CD24 clearly differed in these different tumor cells upon expansion in BME when compared to cells in GD. We conclude that the choice of scaffold utilized for the generation of organoids has an impact not only on cell growth and organoid size but also on protein expression. The disadvantages of batch-to-batch-variations of BME must be balanced with the phenotypic bias observed with GD scaffolds when standardizing organoid cultures for clinical diagnoses.

## 1. Introduction

Overall mortality rates of cancer have declined for some malignancies since the turn of the century [1]. However, diagnosis and therapy of bladder cancer (BC) remain a significant challenge. Bladder cancer is among the most prevalent cancers claiming more than 18 million new cases annually worldwide [2]. However, BC incidence falls in some regions of the world, most likely due to reduced consumption of tobacco, less environmental or occupational pollution, and improvements in diagnosis and especially treatment. BC incidences with 9.6/100,000 in men and mortality rates of 3.2/100,000 in men and 0.9/100,000 women worldwide are still concerning [3]. This means that the need for basic research and more efficient diagnosis and therapy of BC is undiminished. We, therefore, initiated a series of experiments to investigate advanced cell culture methods for analyses of the pathomechanisms contributing to BC initiation and propagation and to generate an alternative in vitro platform to investigate promising new therapies and to facilitate decision-making heading to more effective cancer therapies by individualizing each therapeutic approach.

Cancer research builds on different technologies employing malignant cells and, in many cases, the corresponding healthy counterparts. Classically, these studies are performed in standard cell culture systems using cancer cell lines or by employing different animal models of cancer research. Studies with cancer cells and controls are comparably simple and affordable. However, cell culture systems using cell lines inherit their specific disadvantages, including a clonal bias, lack of cancer cell communication with stromal cells, lack of the contribution of the tumor vasculature, and infiltrating inflammatory cells found in cancers in vivo [4]. Knowledge of animal cancer models expanded our understanding of BC pathology significantly [5,6], but they are comparably expensive and require extra effort and facilities. Depending on the study design and species or even breeds employed, such studies yield in many cases no robust nor translatable results [7]. In addition, animal studies should be reduced to the levels essential for medical or biological research due to ethical concerns [8]. Therefore, alternatives to simple cell culture experiments or animal studies are urgently needed.

Production of organoids gained much interest recently. It is a technology, facilitating the growth of complex blends of cells in defined three-dimensional scaffolds [9,10,11]. Organoids contain stem cells or progenitor cells as well as a variety of other cells found in the tissue samples used for organoid production. In the context of the cell–cell and cell–matrix interactions, the stem or progenitor cells differentiate in the organoids along the corresponding germline lineages to generate a tissue resembling the composition and architecture of the tissue source from which it was derived [11,12,13]. Thus, organoids bridge between in vitro cell culture systems and animal studies and are powerful tools to investigate different biological processes much more closely to the in vivo situation. Organoids were generated from a variety of tissues, including mammary glands, gastrointestinal progenitor niches, or a variety of cancer specimens [11], among them, vesical tissue samples from BC patients [14,15,16,17,18]. Such BC organoids (BCOs) were used for drug screening [18,19,20], investigating the potential of tumor-specific CAR-T cells [21], as a mechanism of tissue vascularization [22], or exchanging of fluids between cells in 3D constructs [23].

The 3D organoid cell culture technology was developed 30 years ago from mouse mammary gland samples and mammary tumors [9]. That study employed a rodent tumor-derived extract as a scaffold containing mainly type IV collagen, laminin-111, and low amounts of other components [9]. Such extracts are commercially available from different providers and are known for instance as Matrigel^®^ or basement membrane extract (BME^®^). Depending on production protocols or batches, these extracts vary in their composition and content of low molecular weight components, such as growth factors, proteases, or enzymes [24,25,26]. The BCOs described some 5 years ago in two early key studies used either Matrigel^®^ [14] or BME^®^ [15] from two different providers. In addition, the cell culture media employed in these first studies differed quite considerably as well [14,15]. However, in at least one of the studies, the composition of the organoid media was disclosed in detail [15]. This facilitated further research and reproduction of the results, but the composition, mechanical properties, and other factors of cell culture scaffolds produced from natural sources, including tumor cell lines, are highly variable and not always disclosed [24,25]. In addition, scaffolds from natural sources cannot be adapted to the specific need of an experiment to the same extent when compared, e.g., to polymer hydrogels [26]. However, for standardizing preclinical studies and in the context of drug screening with cells from individual BC patients, standardized procedures would be preferable. We, therefore, investigated if an animal-free, cellulose-based hydrogel known as GrowDex^®^ (GD), granted the growth of human BCOs.

## 2. Materials and Methods

### 2.1. Culture of Bladder Cancer Cell Line, RT112, in 3D Organoids

The BC cell line RT112 (AC488; available from DSMZ Leibnitz Institute Braunschweig, Germany; a generous gift from Prof. Peter Black, Vancouver Prostate Center) was expanded in DMEM (Thermo Fisher, Waltham, MA, USA) media enriched by 10% FBS and antibiotics as described [27]. To generate spheroids from the tumor line, RT112 cells were harvested with the aid of trypsin-EDTA (Sigma-Aldrich, St. Louis, MO, USA) and washed twice with cold PBS (Sigma-Aldrich, St. Louis, MO, USA). The yield and viability of the cells were determined by trypan blue dye exclusion and a hematocytometer. To generate spheroids, 2 × 10^4^ cells per well were mixed with BME (R&D Systems, Minneapolis, MN, USA) or GD (UMP Biomedicals, Helsinki, Finland), respectively, as described below. The RT112 cells were expanded in spheroids in DMEM media enriched by 10% FBS and antibiotics.

### 2.2. Isolation of Cells from Urothelial Carcinoma Tissue from Bladder Cancer Patients

Tumor tissue samples were obtained after informed and written consent from BC patient # BCO 140. Information on the patients and stages of the disease is summarized in Table 1. The tissue was minced by blade and scissors followed by enzymatic degradation as described recently [17,18]. Debris was removed by passing the extract through a cell strainer with 70 µm mesh size and cells were washed by centrifugation (150 g, 7 min, 20 °C). Yield and viability were enumerated by cell counting in a hematocytometer and trypan blue dye exclusion. The cells were expanded as organoids in OEM as described below. The study was approved by the ethics committee under file number 840/2020BO2 and conducted in full compliance with the WMA Declaration of Helsinki and all other relevant guidelines and regulations.

### 2.3. Isolation of Cells from Rinsing Urine of Bladder Cancer Patients

Rinsing urine samples of patient UCO#33 diagnosed with BC were collected after informed and written consent, cooled on wet ice, and diluted by the addition of equal amounts of PBS. The cells were sedimented by centrifugation (250× *g*, 10 min, 4 °C). Information on the patients and stage of the disease is summarized in Table 1. The sediments were washed twice with washing buffer (PBS, 1% (*v*/*v*) penicillin/streptomycin (Thermo Fisher, Waltham, MA, USA); 0.2% (*v*/*v*) amphotericin B (Sigma-Aldrich, St. Louis, MO, USA) and resuspended in 1 mL media. The yield and viability of cells were assessed by cell counting in a hematocytometer and trypan blue dye exclusion. However, exact numbers of viable cells were hard to determine due to debris in the first fractions of cell preparations from voiding urine samples.

### 2.4. Expansion of Cells in Organoid Cultures

The initial cell density utilized for the generation of organoids from BC voiding and tissue samples was 2 × 10^6^ cells/mL. For the generation of organoids, 10 μL of the cell suspension corresponding to 2 × 10^4^ viable cells total were mixed on wet ice with 30 μL of the BME hydrogel stock (R&D Systems, ≈15 mg/mL), either depleted of growth factors (type 2; BME−) or containing growth factor (BME+) and mixed by gentle pipetting. Two aliquots of 20 μL each were dropped in a well of a 48-well culture plate. The plate was turned over and incubated at 37 °C for 15 min in a humidified cell culture incubator to jell the hydrogel containing the cells. Then, the 48-well plate was turned back, and the hydrogel domes were covered by 250 μL of organoid expansion medium (OEM) per well [15]. The OEM contained 22.2 mL advanced DMEM-F12 (Thermo Fisher) and 22.5 mL of wnt-, R-spondin-, and noggin media (generously provided by Dr. André Koch, Dept. of Gynecology, UKT@EKUT) [28] and was enriched by 2.5 mL 5% charcoal-stripped FBS, 1 mL B27-supplement, 500 μL L- glutamine, 500 μL HEPES (1 M), 500 μL nicotinamide (1 M), 125 μL N-acetylcysteine (500 mM), 50 μL A83-01 (5 mM), 50 μLl Primocin (50 mg/mL), 50 μL FGF-10 (100 μg/mL), 25 μL FGF-7 (50 μg/mL), 12,5 μL FGF-2 (50 μg/mL), 5 μL Y-27632 (100 mM), and 0,5 μL EGF (500 μM) (all from Sigma-Aldrich or PeptroTech, Rehovot, Israel) [15]. Organoids were cultured in an incubator at 37 °C in 5% CO_2_ and a humidified atmosphere. OEM was changed two to three times per week depending on cell density, integrity, and size of the organoids. To isolate cells from BME hydrogels, OEM was aspirated, and the scaffold was degraded enzymatically by adding 250 μL prewarmed PBS and 50 μL dispase (Roche) to the well followed by a 60 min incubation at 37 °C in a humidified cell culture incubator. Then, 200 μL of trypsin-EDTA (Sigma-Aldrich) were added per well, mixed carefully, transferred in a 2 mL tube, and incubated in a thermomixer (Eppendorff; 37 °C, 1400 rpm, 17 min). After proteolytic degradation of the scaffold, the suspension was aspirated and mixed with 1 mL OEM, and the cells were sedimented by centrifugation (150× *g*, 5 min, 20 °C). Supernatants were removed, the sediment was chilled on wet ice, and the cells were counted as described above. Fresh domes were generated from 2 × 10^4^ cells as described above, or cells were used for other experiments.

To generate spheroids or organoids with an animal-free cellulose-based hydrogel, different dilutions of the original stock (i.e., 1.5% GrowDex^®^ (GD), UMP Biomedicals, Helsinki, Finland) were prepared with OEM ranging from 0.2% to 1% and tested with RT112 cells for cell density and growth kinetics. Based on the outcome of the preparatory tests with RT112 in GD scaffolds, dilutions of 0.2% and 0.5% GD in OEM and an inoculation density of 5 × 10^4^ cells per well were used in the study. To isolate the cells from the GD hydrogel, the enzyme GrowDase^®^ was used as recommended by the supplier (UMP Biochemicals). After degradation of the scaffold, the cells were washed twice with OEM, counted, and either expanded as organoids or used for experiments.

### 2.5. Freezing and Thawing of Organoids

The cells were harvested from organoids as described above, resuspended in 500 µL OEM in a cryotube on wet ice, mixed with 500 µL ice-cold freezing medium (20% DMSO (AppliChem, Darmstadt, Germany), 30% FCS (Sigma-Aldrich), and 50% RPMI 1640 (Gibco Life Technologies, Waltham, MA, USA)), chilled slowly to −80 °C overnight, and then transferred to a liquid nitrogen tank for long-term storage. To thaw organoids, cryotubes of liquid nitrogen were transported on wet ice to the 37 °C water bath. The cells were thawed, transferred very quickly in 10 mL OEM, mixed gently, and sedimented by centrifugation (150× *g*, 5 min 20 °C). The domes were generated as described above, or cells were used for other experiments.

### 2.6. Immunofluorescence of Spheroids and Organoids and Fluorescence Microscopy

To detect the expression of BC marker genes or lineage markers on UCO#33, BCO#140, and RT112, immunofluorescence on chamber slides was applied. The spheroids or organoids were isolated from the hydrogel as described above (in the case of BME without the trypsin-EDTA step), dissolved in OEM complemented by 3.3% BME, transferred in 8-well chamber slides (x-well cell culture chamber, on PCA slide; Sarstedt), incubated for 2 h (37 °C, 5% CO_2_), and fixed by 4% formaldehyde (30 min, 20 °C) before staining. Then, the spheroids or organoids were washed three times with PBS, blocked (5% BSA (Sigma-Aldrich), 0.2% Triton X-100 (Merck, Rahway, NJ, USA), 0.1% Tween 20 (Sigma-Aldrich) in PBS, 30 min, 37 °C), washed with PBS again, and incubated with primary antibodies (1 h, 37 °C, humidified chamber, dark, Table 2). Unbound antibodies were washed away with PBS (3 × 5 min, slow orbital shaker, 20 °C). The samples were incubated with fluorescence-labeled secondary antibodies (1 h, 37 °C, humidified chamber, dark; Table 2). Unbound secondary antibodies were washed away with PBS (see above). Cell nuclei were stained with DAPI. The antibody diluent was 1% BSA in PBS. Samples were observed by fluorescence microscopy (Axiophot; Zeiss, Jena, Germany) equipped with an AxioCam MRm camera (Zeiss), a UV light source (Leica EL6000, medium power), and, unless otherwise stated, by 40× objective. Micrographs and fluorescence overlays were generated using proprietary software (AxioVision 4.8, Zeiss). Whole organoids were recorded as projection images. Additionally, staining of organoids was performed with secondary antibodies as the control and to determine detection levels (Figure S1).

### 2.7. Statistics

The experimental data were processed by spreadsheet software (Excel^®^, Microsoft, Albuquerque, NM, USA) and tested for normal distribution (skewness, kurtosis, Kolmogorov–Smirnov test, Shapiro–Wilk-test) and in the histogram with normal distribution curve using a statistic program (SPSS Statistics; IBM, Endicott, NY, USA). A normal distribution was not found in any of the groups so the medians and non-parametrical Mann–Whitney U-test were employed for the comparison of the groups. However, the Mann–Whitney U-test for the comparison of GD 0.5% and BME– is unlikely to be meaningful due to the small number of samples in the cohort GD 0.5% (*n* = 7).

## 3. Results

### 3.1. Optimizing Scaffold Concentrations for Bladder Cancer Cell Cultures

**Figure 1:**
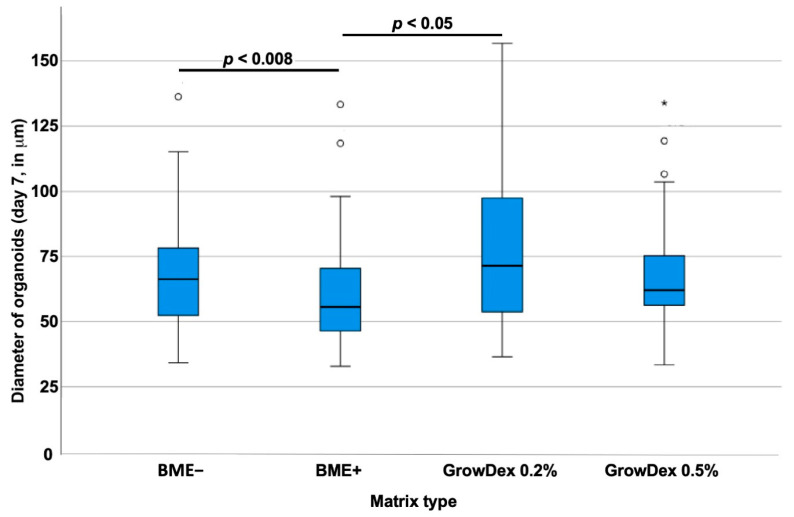
Size of RT112 organoids in BME in comparison to GD scaffolds. RT112 were seeded in scaffolds generated by cytokine-deprived (BME−) or cytokine-enriched (BME+) Matrigel@ or in GD at 0.2% or 0.5% (*v*/*v*) density as indicated (*x*-axis). After seven days of 3D culture, the sizes of organoids were determined by microscopy. The median diameter of organoids is depicted on the *y*-axis (scale in mm). Outliers are displayed as dots or stars. Abbreviations: BME, basal membrane extract.

Most studies exploring urothelial carcinoma, e.g., for cancer therapy testing in three-dimensional (3D) culture systems, employed Matrigel^®^ or BME in either growth factor-depleted (BME−) or growth factor-containing (BME+) formulation. However, the attachment of cells to a matrix and the complementation of cell culture with growth factors influence the cell’s metabolism. We, therefore, investigated the effects of GD in two different concentrations on the size of RT112 spheroids in comparison to BME− and BME+ respectively (Figure 1).

Normal distribution of data was not found. A Kruksal–Wallis test revealed significant differences in the median spheroid sizes between at least two of the four matrix-type groups investigated (*p* < 0.008). The RT112 generated in BME− significantly larger spheroids (median diameter 66.31 ± 19.8 mm; *n* = 84) when compared to cells in BME+ (median diameter 55.65 ± 20.9 mm; *n* = 45; *p* < 0.05). Thus, cell growth of this tumor line was facilitated in BME− when compared to BME+, despite lower concentrations of growth factors (Figure 1). In 0.2% GD, larger RT112 spheroids were observed in comparison to BME+, (median diameter 71.44 ± 28.3 mm; *n* = 48; *p* < 0.05) but not in comparison to BME− (*p* = 0.06). In a 0.5% GD scaffold, the sizes of RT112 spheroids were not significantly different in comparison to 0.2% GD (median diameter 62.07 ± 20.5 mm; *n* = 49; *p* = 0.087). As GD is not complemented by cytokines, the role of the scaffold for growth RT112 in three-dimensional spheroids is emphasized.

**Figure 2:**
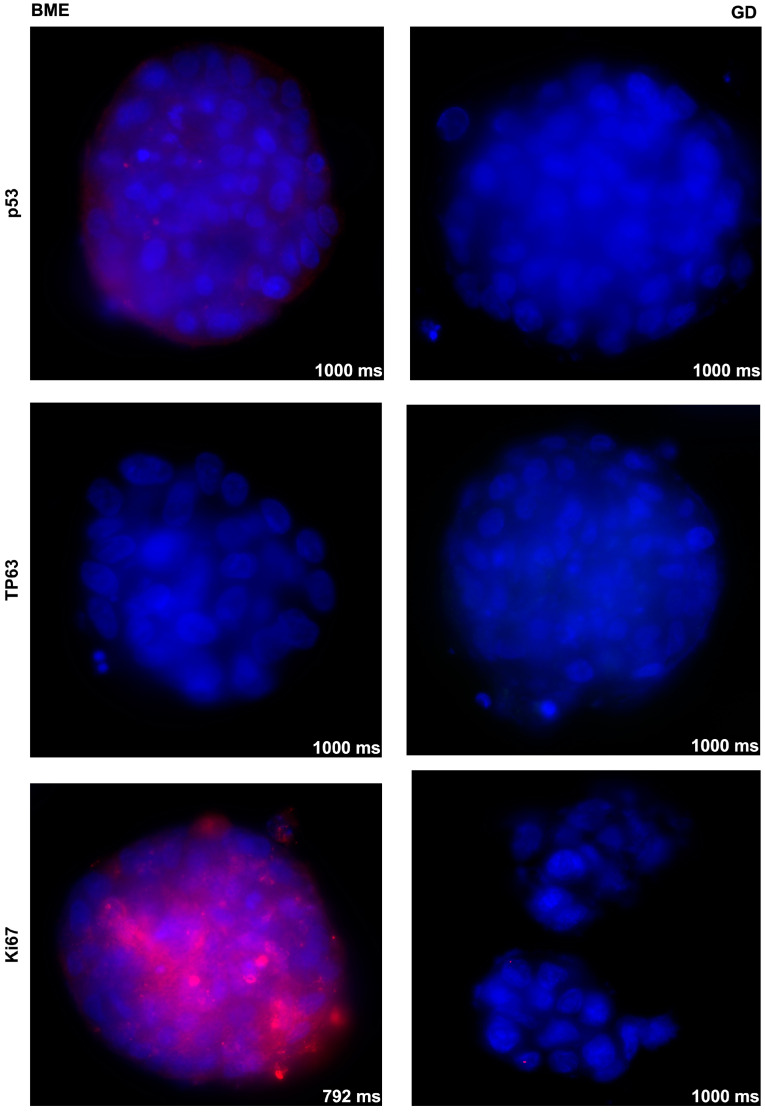
Analysis of marker gene expression in RT112 spheroids as a function of the scaffold composition. The expression of p53 (**top**), TP63 (**middle**), and Ki67 (**bottom**) in RT112 cells cultured in BME− (**left**) or 0.2% GD (**right**) was investigated by immunofluorescence and labeled in red. Cell nuclei are indicated in blue (DAPI). The exposure time is indicated in the individual micrographs in milliseconds, objective 40×. Control stainings omitting the primary antibody are provided in the supplement. Abbreviations: BME, basal membrane extract; GD, GrowDex^®^.

In addition, the expressions of proliferation marker Ki67, tumor-associated tumor suppressor p53, and transcription factor TP63 were investigated by immunofluorescence in spheroids generated by RT112 in BME− in comparison to 0.2% GD. Expression of Ki67 was recorded in RT112 in BME− but not in RT112 in 0.2% GD (Figure 2), while TP63 and p53 remained below detection levels in RT112. This indicated that the choice of scaffold had an influence on gene expression levels, even in urothelial cancer cell lines.

### 3.2. Effects of Basement Membrane Extract- Versus Cellulose-Derived Scaffolds on Urothelial Carcinoma-Derived Organoid BCO#140

**Figure 3:**
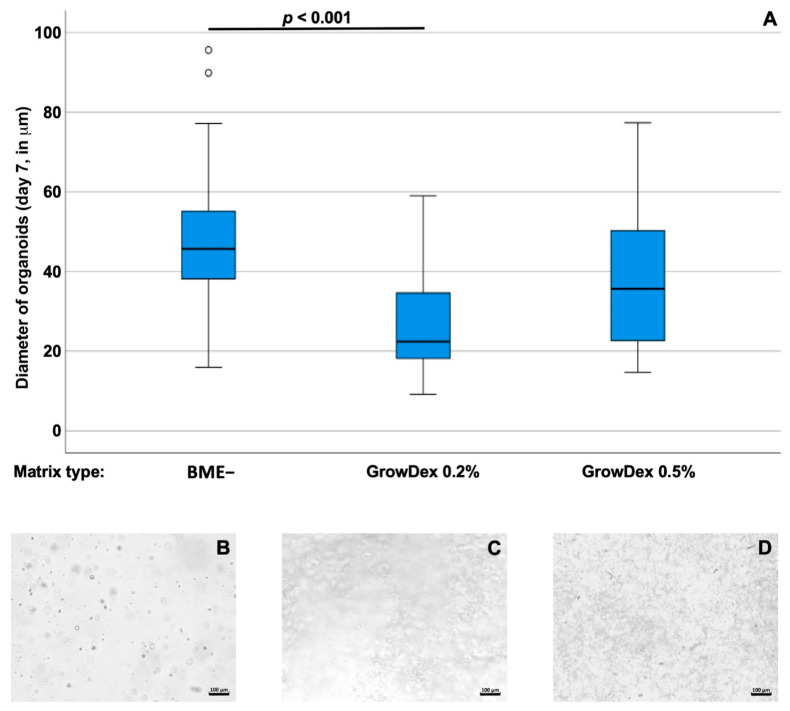
(**A**) Size of bladder cancer tissue-derived organoids BCO#140 in BME− scaffolds in comparison to GD at 0.2% or 0.5% (*v*/*v*) scaffolds as indicated (*x*-axis). After 7 days of 3D culture, sizes of organoids were determined by microscopy. The median diameter of organoids is depicted on the *y*-axis (scale in mm). Outliers are displayed as dots. Abbreviations: BME, basal membrane extract. (**B**–**D**) Examples of overview micrographs of BCO#144 in BME− (**B**), GD 0.2% (**C**), or DG 0.5% (**D**), respectively. Size bars indicate 100 mm.

Bladder cancer tissue-derived organoids BCO#140 were seeded in BME−, 0.2%, and 0.5% GD scaffolds, respectively, and incubated for 7 days in culture (Figure 3). For BCO#140, significantly larger organoids were found in BME− (median diameter 45.7 ± 14.6 mm; *n* = 80) when compared to organoids in 0.2% GD (median diameter 22.4 ± 14.1 mm; *n* = 23, *p* < 0.001), while organoids seeded in 0.5% GD (median diameter 35.7 ± 22.2 mm; *n* = 7, *p* = 0.17) were not significantly different in size when compared to BCO#140 in BME− or 0.2% GD (Figure 3).

**Figure 4:**
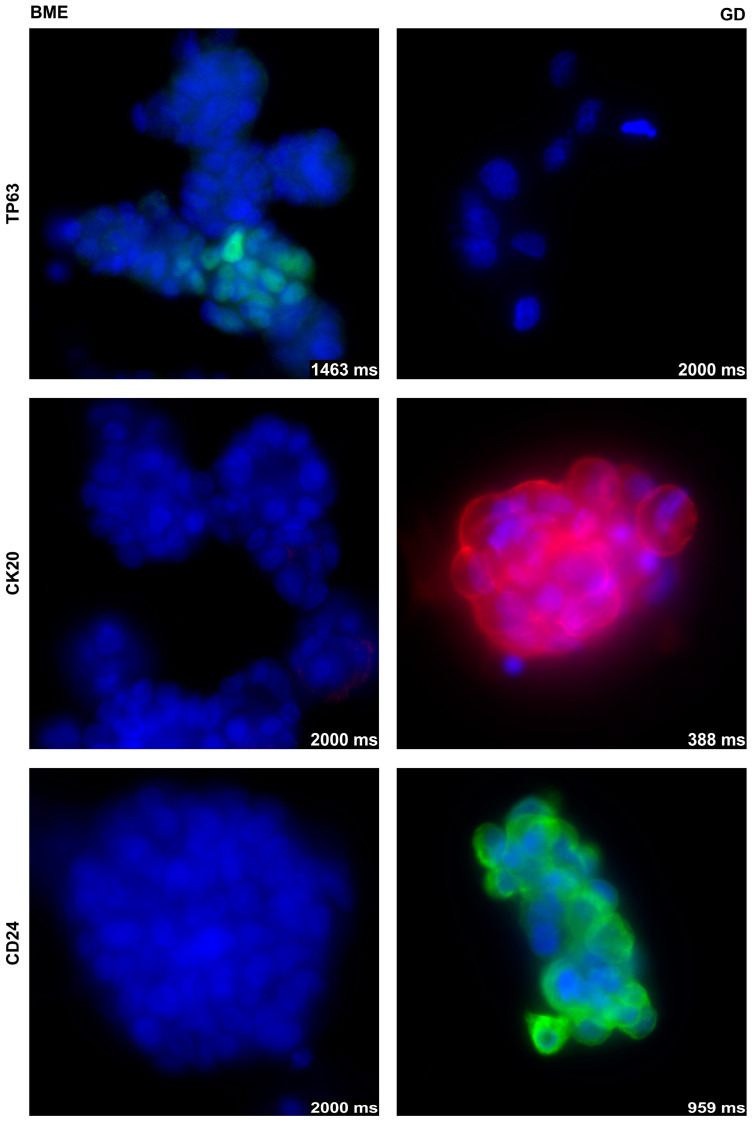
Expression of TP63 (**top**), CK20 (**middle**), and CD24 (**bottom**) in BCO#140 cultured in BME− (**left**) or 0.2% GD (**right**) scaffolds. A moderate expression of TP63 was detected in BCO#140 organoids in BME− but not upon even extended exposure on BCO#140 in GD. Expression of CK20 and CD24 was found in BCO#140 organoids in 0.2% GD but not even after extended exposure in BCO#140 in BME−. The exposure times in milliseconds (ms) are included in each micrograph, objective 40×. Control stainings omitting the primary antibody are provided in the supplement. Abbreviations: BME, basal membrane extract; GD, GrowDex^®^.

In addition, the expression of some representative bladder tumor-associated markers was investigated by immunohistochemistry in organoids of BCO#140 expanded in BME− or 0.2% GD (Figure 4). Expression of TP63 was detected in BCO#140 in BME− but not in GD, even after extended exposure (Figure 4). In contrast, the expression of cytokeratin 20 (CK20) as well as the expression of CD24 were recorded in BCO#140 cultured in GD but not in BME−, not even after extended exposure (Figure 4). This result corroborated the effects observed in RT112 spheroids (Figure 2). We conclude that the composition of the scaffold influences the metabolism of bladder cancer tissue-derived cells.

### 3.3. Effects of Basement Membrane Extract- Versus Cellulose-Derived Scaffolds on Urine-Derived Organoid UCO#33

**Figure 5:**
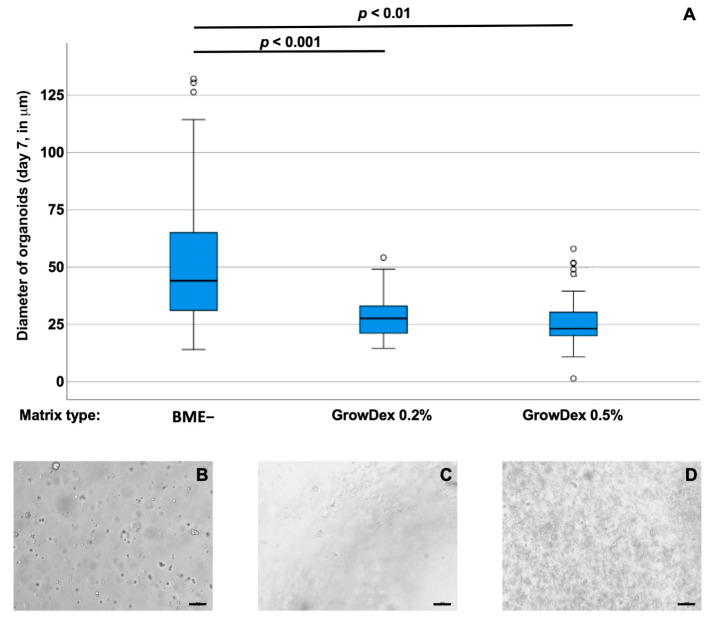
(**A**) Size of urine-derived organoids UCO#33 in BME− scaffolds in comparison to GD at 0.2% or 0.5% (*v*/*v*) scaffolds as indicated (*x*-axis). After 7 days of 3D culture, sizes of organoids were determined by microscopy. The median diameter of organoids is depicted on the *y*-axis (scale in mm). Outliers are displayed as dots. Abbreviations: BME, basal membrane extract. (**B**–**D**) Examples of overview micrographs of UCO#33 in BME− (**B**), GD 0.2% (**C**), or DG 0.5% (**D**), respectively. Size bars indicate 100 mm.

**Figure 6:**
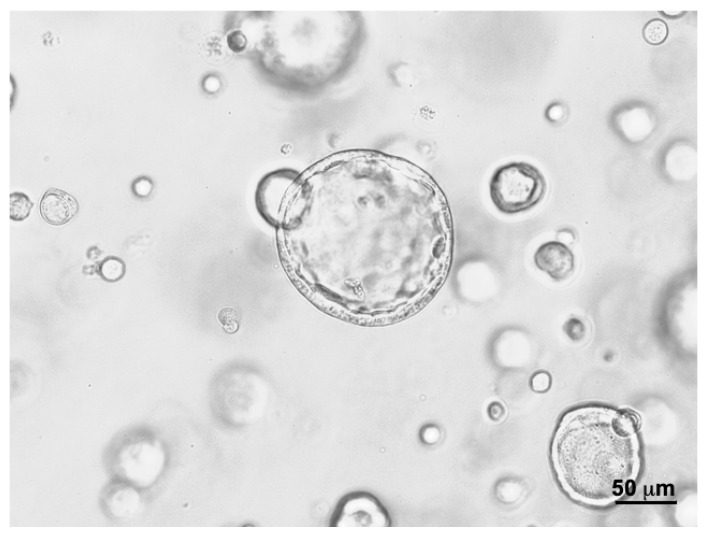
Organoids of UCO#33 appeared in BME− in early passage cultures as spheroids. The cell density seemed less pronounced in the centers of the spheroids when compared to the outer rim. The size bar indicates 50 µm.

Urine samples were collected from a patient diagnosed with bladder cancer to isolate cells for the generation of urine-derived cancer organoids (UCOs). In 77% of all urine-derived cultures generated (27/35), organoids were observed in primary cultures. Cells from UCO#33 (parental tumor size 23 × 15 × 10 mm) were seeded in BME−, 0.2%, or in 0.5% GD scaffolds, respectively, and incubated for 7 days in culture (Figure 5). For UCO#33, significantly larger organoids were found in BME− (median diameter 44.05 ± 25.3 mm; *n* = 205) when compared to organoids in 0.2% GD (median diameter 27.71 ± 9.3 mm; *n* = 36, *p* < 0.001) or when compared to organoids in 0.5% GD (median diameter 23.2 ± 9.6 mm; *n* = 83, *p* < 0.01). Differences between UCO#33 in 0.2 versus 0.5% GD were not recorded (Figure 5). These results are in clear contrast to the observation with cell line RT112 (Figure 1) but in line with the results observed with BCO#140 (Figure 3) as BME− facilitated the growth of larger organoids from cells isolated from clinical samples and expanded in OEM medium in organoid cultures (Figure 5). A representative micrograph of UCO#33 in culture is shown in Figure 6.

**Figure 7:**
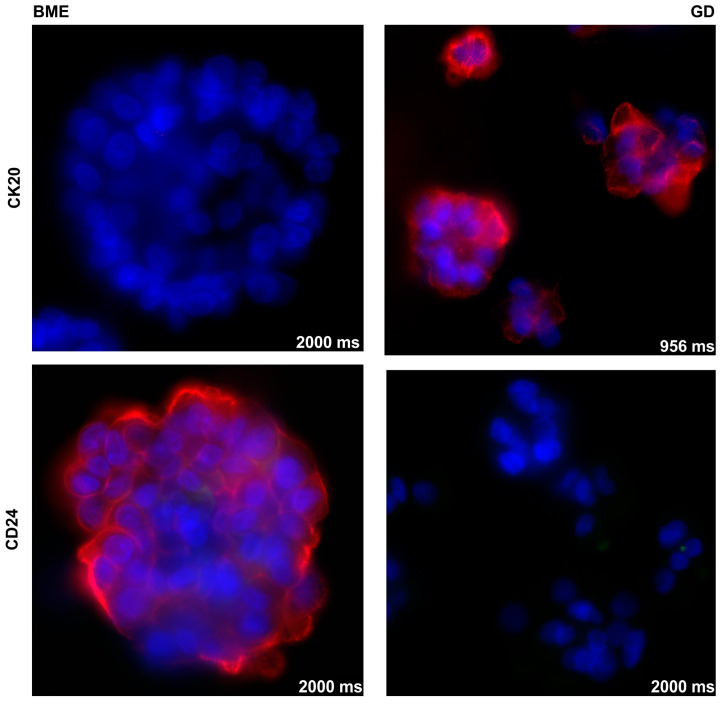
Expression of CK20 (**top**) and CD24 (**bottom**) in UCO#33 organoids cultured in BME− (**left**) or 0.2% GD (**right**) scaffolds. Expression of CK20 was found in UCO#33 in 0.2% GD but not in cells in BME−, not even after extended exposure. In contrast, low expression of CD24 was detected in UCO#33 upon expansion in BME− but not in cells in GD. The exposure times in milliseconds (ms) are included in each micrograph, objective used 40×. Control stainings omitting the primary antibody are provided in the supplement. Abbreviations: BME, basal membrane extract; GD, GrowDex^®^.

The expression of some representative bladder tumor-associated markers was investigated by immunohistochemistry on UCO#33 in early passage in BME−cultures (Figure S2). Intensive yellow staining provided evidence for a prominent co-expression of tumor marker GATA-3 and the AE1/AE3 reactive cytokeratins on UCO#33 by immunofluorescence (Figure S2A) as well as co-expression of bladder tumor-associated antigens CD24 and of CD44. However, on some organoids, only a weak expression of CD24 but no CD44 was found (Figure S2B). The proliferation marker Ki67 was detected in virtually all cells of UCO#33 (Figure S2C), and some, but not all, cells expressed the epithelial or urothelial marker CK 7 (Figure S2D). This suggested that at least some cells contained in UCO#33 resembled marker expression patterns associated with bladder cancer cells. BCO#33 was expanded in BME− versus 0.2%GD as well (Figure 7). Expression of TP63 was not detected in UCO#33 (not shown), but the expression of the epithelial or urothelial marker cytokeratin 20 (CK20) was recorded in UCO#33 cultured in GD but not in BME−, not even after extended exposure (Figure 7). The expression of CD24 was weak in BME− and recorded by extended exposure but not detected in UCO#33 organoids in GD (Figure 7). This corroborated that the composition of the scaffold has an effect on the phenotype of BCOs and UCOs in culture.

## 4. Discussion

Three-dimensional (3D) cultures of cells, such as spheroids and organoids, have gained significant interest in current discussions. While spheroids refer to multicellular and 3D cultures without specifying the blend and type of cells, in this study we stick to the definition of organoids as 3D cell cultures containing—besides a scaffold—cells of different lineages and differentiation stages and especially differentiation and proliferation competent stem cells and/or progenitor cells. The 3D cell cultures of established cell lines, such as RT112 and others, are therefore considered spheroids [29], while cultures containing a blend of cells isolated from healthy or diseased tissues and seeded in suitable hydrogels are regarded as organoids. Such organoids resemble the in vivo situation of tissues or tumors much better when compared to standard 2D cultures [30]. However, vascularization and hormonal or neuronal signals may not be provided in organoids to the extent observed in animal models or real clinical situations. This of course inherits several challenges when tumor models are needed to investigate optimal or novel therapies for individual cancer patients.

In aiming at technologies to establish robust protocols for future tests in search of efficient therapies, patient-derived organoids were employed [14,18,19,20], and significant differences between 2D and 3D cultures were observed [18]. Mechanisms of drug action may here come into account. For instance, cisplatin or its derivatives are complexes of platinum with different ligands, which bind to purine residues of DNA [31]. Upon diffusion across the cell membrane and in the nucleus of cells, cisplatin blocks DNA replication and induces apoptosis [31]. Thus, it acts on faster-proliferating cells but is of course not tumor specific. However, limitations of cisplatin diffusion in 3D constructs when compared to cells in flat 2D cultures may bias dose-response curve assays screening drug efficacies at short incubation times. Therefore, the significant differences in organoid size—and possibly in the density of cell clusters in a dome—should be considered when investigating tumor sensitivities in organoids using rapid throughput systems. Remarkably, the matrix density of GD did not cause significant differences in spheroid nor organoid densities, but significant differences were observed between the scaffolds included, i.e., BME versus GD.

Cells binding in vitro to pericellular components or scaffolds and in vivo to the different hydrogels may therefore not be compared without critical consideration. Integrin-binding peptide motives were defined on type IV collagen and laminin-111, the main components of Matrigel^®^ or BME [32,33]. Cells therefore can interact directly with such scaffolds. In contrast, direct binding of cells to cellulose seems unlikely. However, proteins in OEM may adhere to the cellulose scaffold and thus facilitate an indirect attachment of the cells to the hydrogel. Integrin binding activates the focal adhesion kinase, which in turn activates an anti-apoptotic situation in cells [34]. The scaffolds used for the generation of 3D cultures may therefore bias cancer drug assays. Moreover, efficient binding of cells via integrins requires their activation by divalent cations [35,36] and TGF-b [37]. As the OEM containing the TGF-signaling blocker A83-01, enhanced integrin signaling seems not to influence the cell viability [34], and significant differences in cell survival were not recorded in cells in BME when compared to GD (not shown). However, integrin signaling modulates the apoptotic response of cells to cisplatin- or doxorubicin-induced cell death [38].

However, non-integrin receptors for type IV collagen and laminin-111 are known as well. In contrast to integrins, they do not need to change their configuration to an active and erected matrix-binding heterodimeric complex. For instance, the adhesion molecule CD44 is a glycoprotein and binds to type IV collagen [39]. Expression of CD44 was associated with tumor metastasis [40,41]. Elevated expression of CD44 was reported on bladder cancer tissue samples, primary cells, cell lines, and organoids [17,42]. Moreover, the discoidin domain receptor 1 (DDR1, alias CD167) is another example of an integrin-independent receptor for type IV collagen [43]. DDR1 is a tyrosine kinase involved in the regulation of cell growth and metabolism. Expression of DDR1 is described on different epithelial cells; its overexpression in bladder cancer was associated with poor outcomes [44]. The same applies to laminin-111-mediated cell attachment. A monomeric 67 kDa receptor for laminin-111 was described on mouse tumor cells as early as four decades ago [45]. This 67 kDa was detected on human breast, prostate, and colon cancer cells [46,47]. Thus, integrin-independent attachment of cancer cells to hydrogels in organoids is granted, but their role in the context of 3D cell culture spheroids or organoids remains to be explored.

To target bladder cancer tumors more specifically, cell surface antigens, such as fibroblast growth factor receptor 3, CD276, CD47 [48,49,50,51,52], or intracellular antigens, such as p53, TP63, isoforms of the Pi3-kinase, hRas, her2, and others, were considered [53,54]. Furthermore, differentiation into pathological or molecular subtypes of bladder cancer, such as basal (CK5/6 positive, CK20 negative) and luminal-like expression profiles, hold the potential to predict beneficial responses towards, e.g., neoadjuvant chemotherapy, which provided the best survival benefit among the basal subtype [55]. Here, expression levels of these antigens on and within the cells take the spotlight of interest both in histopathological evaluation and in the culture systems as individual features of each tumor should be preserved in a potential prediction and screening model. In our investigations, we found controversial results regarding the expression of CK20 depending on the scaffold used. In both organoids investigated, BCO#140 as well as UCO#33, GD promoted a CK20 positive luminal-like subtype, while cultivation of an autologous specimen in BME led to a CK20 negative, basal like-subtype. The relevance of this finding should be evaluated in a larger cohort size; however, it is crucial to consider the potential impact of the scaffold on the molecular subtype when using organoids as drug-screening models to guide personalized therapeutic approaches. The role of CD24 is highlighted by the overexpression on different tumors, and knock-down of CD24 expression revealed its function in tumor cells: CD24 depletion reduced cell proliferation, enhanced sensitivity to undergo apoptosis, and modulated STAT3-mediated gene expression [56]. Moreover, CD24 was reported as a predictor of bladder cancer recurrence [57], and recent proof-of-principle studies provided promising results of anti-CD24 cancer therapy. However, the results for proof of the efficacy of anti-CD24 therapies are limited [58]. We showed that the expression of CD24 differs in BCOs or UCOs depending on the scaffold used. Therefore, the efficacies of drugs targeting cell surface structures will yield different outcomes depending on the experimental design employed.

Investigation of the complex differences in gene regulation of cells as a function of the scaffold is beyond the topic of this study. Due to the expected limitations of materials available, an in-depth investigation of gene expression on, e.g., transcript versus protein versus cell surface levels, was not intended. We noted, however, that the very same cells, be that a cell line such as RT112, tumor cells BCO#140 isolated from a solid tumor, or UCO#33 cells from rinsing urine samples—behave differently in BME versus GD. Elevated expression of proliferation marker Ki67 in RT112 in BME seems most likely a matrix-induced difference and to a subordinate extent modulated by growth factors as no difference in organoid sizes was recorded between BME+ in comparison to BME− cultures. In BCO#140, BME− induced expression of TP63, while CK20 and CD24 were elevated in GD organoids. As growth factor-depleted BME− was employed in these experiments, major differences in the growth factor contents of the cultures are less pronounced. Here, the role of the matrix, its structure and complexity, and the density of domains or peptide motives for cell attachment seem more important. The same was observed with UCO#33, where CK20 was elevated in GD cultures, while CD24 was prominent in BME− organoids. However, a general trend indicating which gene is expressed at elevated or reduced levels cannot be deduced from this small proof-of-principle study as the impact of patient-interindividual effects could not be studied. Serial investigations of larger cohorts of BCOs and UCOs will facilitate discrimination between the influence of the individual donor on the assay and the effects of the scaffold materials used. However, when screening for sensitivities of cancer cells for treatment efficacy, the bias of the scaffold should be considered.

## 5. Conclusions

Organoids derived from solid tumor tissue and from voiding urine samples of bladder cancer patients can be propagated and studied in 3D cultures complemented by both collagen- and laminin-containing Matrigel^®^ or BME^®^ scaffolds as well as cellulose-based GrowDex^®^ hydrogels. However, significant differences in the sizes of cell clusters were recorded, and distinct patterns of gene expression patterns were observed. The choice of the scaffold may therefore influence the outcome of a study when standardized assays are developed for drug screens with cells of individual donors in preparation for an efficient regimen.

## Figures and Tables

**Table 1:** Clinical data on patients # UCO33 and # BCO140. Abbreviations: pT, pL, pV, pathological staging (p) of the tumoral (T), lymphatic (L), and venous invasion of the tumor cells; CIS, carcinoma in situ; BCO, tissue-derived bladder cancer organoid; UCO, urine-derived bladder cancer organoid; n.a., not applicable.

	BCO#140	UCO#33
Sex	male	male
Age	62	85
Pathology		
pT	1	a
pL	1	n.a.
pV	1	n.a.
Grading	high grade	low grade
CIS	yes	no
Urine cytology	positive	positive

**Table 2:** Primary and fluorescence-labeled secondary antibodies employed.

Target	Dilution	Clone	Company
AE1/AE3 antigens	1:200	AE1/Ae3	Merck
CD24	1:100	PR19925	Abcam
CD44	1:200	MEM-263	Abcam
CK7	1:300	EPRY1619Y	Abcam
CK20	1:100	XQ1	Merck
GATA-3	1:100	serum	Abcam
Ki67	1:100	ARG57562	Biomol-Argio
p53	1:100	PAb 240	Invitrogen
TP63	1:100	10H7L17	Invitrogen
gt-a-ms IgG ^1^	1:1000	serum	Jackson Immuno Res.
gt-a-rb IgG ^2^	1:1000	serum	Jackson Immuno Res.

^1^ Cy3- or Alexa488-labelled goat-anti-mouse total IgG; ^2^ Cy3- or Alexa488-labelled goat-anti-rabbit total IgG.

## Data Availability

The data of this study will be made available to all colleagues from public institutions dedicated to research and education upon justified request.

## Supplementary Materials

The following supporting information can be downloaded at: https://www.mdpi.com/article/10.3390/cells12162108/s1, Figure S1: Staining of organoids with secondary antibodies as controls; Figure S2: Detection of bladder cancer markers on UCO#33 by immunofluorescence.
