# Supplementary material for: Effects of Scaffolds on Urine- and Urothelial Carcinoma Tissue-Derived Organoids from Bladder Cancer Patients
Source: Cells. 2023 Aug 20;12(16):2108. doi: 10.3390/cells12162108 (PMC10453567; doi:10.3390/cells12162108)
Supplement: Supplementary file 1 [file cells-12-02108-s001.zip › cells-2517946-supplementary.pdf]

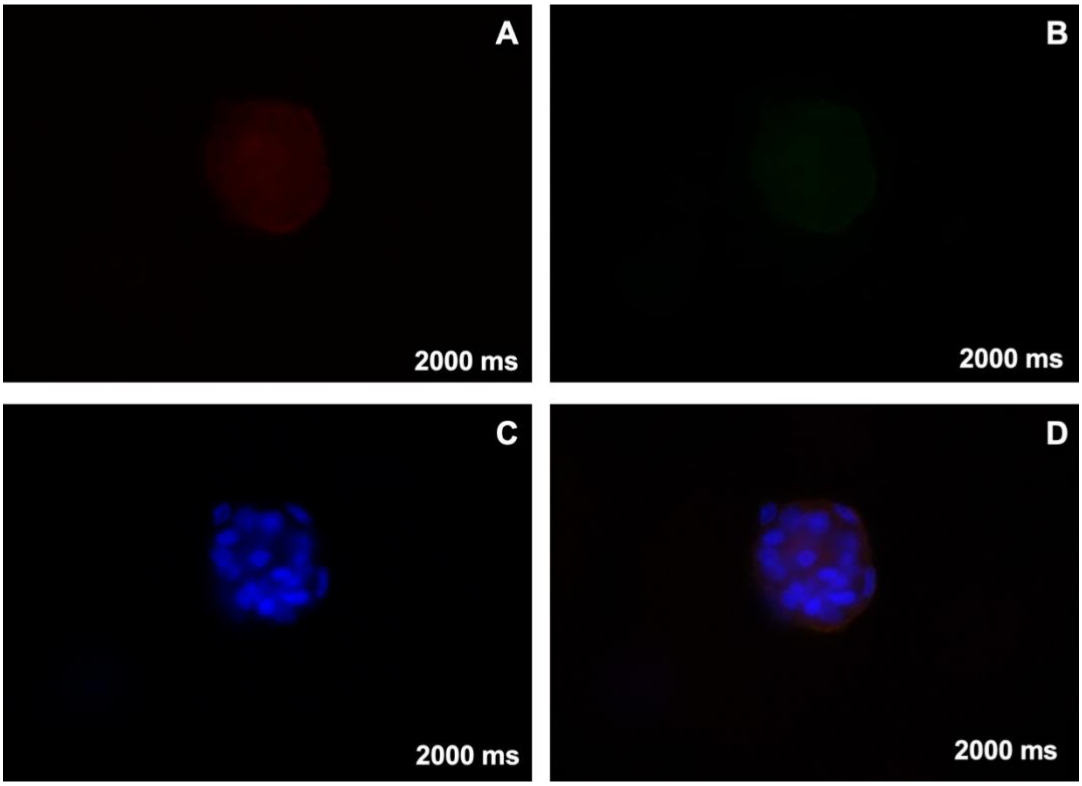

Figure S1. *Staining of organoids with secondary antibodies as controls.*
Organoids UCO#33 were incubated with the detection serum Cy-3-labeled goat-anti-mouse total immunoglobulin (1:1000) or Alexa488-labelled goat-anti-rabbit total immunoglobulin. Micrographs were exposed for 2 seconds as indicated. The micrographs present (A) Cy-3 staining in the red channel, (B) Alexa488-staining in the green channel, (C) nuclear DAPI staining in the blue staining, and (D) the merged pictures combining the three fluorescence channels employed. The goat-anti-mouse antibody showed a minor signal (A) that was considered as the control in comparison to the staining with primary antibodies (see Figs. 7, S2).

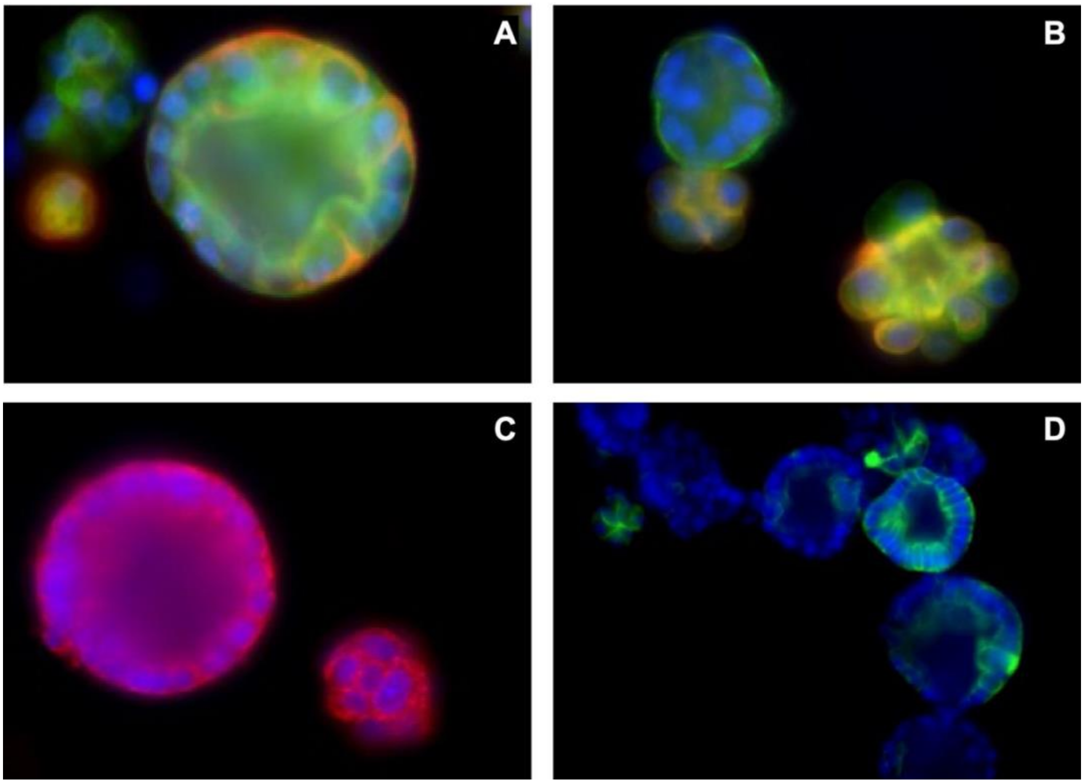

Figure S2. *Detection of bladder cancer markers on UCO#33 by immunofluorescence*

(A) Expression of bladder cancer-associated antigens GATA-3 (green, exposure 350 ms) and pan-cytokeratin AE1AE3 (red, exp. 870 ms) was investigated on UCO#33 expanded in BME-. Some cells expressed GATA-3 only while the yellow fluorescence of other cells indicated double-positive cells or fluorescence overlay on other areas of the micrograph. (B) Expression of bladder cancer-associated antigens CD24 (green, exp. 660 ms) and CD44 (red, exp. 260 ms) were detected on UCO#33 as well. Again, some cells expressed CD24 only while yellow fluorescence indicated co-expression CD24 and CD44 or an overlay of the fluorescence. (C) Virtually all UCO#33 cells express the proliferation marker Ki67 (exp. 410 ms) (D) Expression of the urothelial marker cytokeratin 7 was recorded on some UCO#33 organoids (right side), while it was not detected on others (upper left side, exp. 1720 ms). Counterstaining with DAPI (blue) served to visualize cellular nuclei.
